# Attitudes and impressions of participants in a study of the causes of childhood cancer

**DOI:** 10.1054/bjoc.2000.1578

**Published:** 2001-02

**Authors:** C M Jenkinson, K M Muir, P G Hawtin, C E D Chilvers

**Affiliations:** Division of Public Health Medicine and Epidemiology, University of Nottingham Medical School, Queen's Medical Centre, Nottingham, NG7 2UH, UK

**Keywords:** epidemiology, attitudes, ethics, participation, childhood cancer

## Abstract

Researchers and ethics committees are increasingly concerned about the perceived emotional impact on individuals following participation in epidemiologic studies. This attitudinal survey was designed to investigate this issue among 751 of the parents who had already given an interview in the UK Childhood Cancer Study (UKCCS), one of the largest case-control studies ever undertaken to investigate the aetiology of cancer in children. Information was collected by postal questionnaire on their reasons for agreeing to take part in the UKCCS, on whether questions had caused distress or difficulty and what their feelings were immediately after the interview and at the time of this survey. Parents were asked if they felt they had benefited in any way by taking part and control parents were asked if they would have taken part without prior consent of their doctor. 90% of both cases and controls felt glad to have taken part immediately after the interview and few reported any anxiety at having done so; 95% of both cases and controls felt satisfied that they had made a worthwhile contribution. Although 18% of cases felt tense and 14% felt unhappy after the interview, over 90% of them felt glad that they had taken part a few weeks later. Of particular interest is that 38% of cases and 24% of controls said they had positively benefited from taking part in the UKCCS and 96% of control mothers did not mind their family doctor giving permission for them to be contacted. © 2001 Cancer Research Campaign http://www.bjcancer.com


*British Journal of Cancer* (2001) **84**(3), 413–416
© 2001 Cancer Research Campaign
doi: 10.1054/ bjoc.2000.1578, available online at http://www.idealibrary.com on IDEAL®

http://www.bjcancer.com

### Attitudes and impressions of participants in a study of the causes of childhood cancer

**CM Jenkinson, KM Muir, PG Hawtin and CED Chilvers**

Division of Public Health Medicine and Epidemiology, University of Nottingham Medical School, Queen's Medical Centre, Nottingham, NG7 2UH, UK

**Summary** Researchers and ethics committees are increasingly concerned about the perceived emotional impact on individuals following participation in epidemiologic studies. This attitudinal survey was designed to investigate this issue among 751 of the parents who had already given an interview in the UK Childhood Cancer Study (UKCCS), one of the largest case-control studies ever undertaken to investigate the aetiology of cancer in children. Information was collected by postal questionnaire on their reasons for agreeing to take part in the UKCCS, on whether questions had caused distress or difficulty and what their feelings were immediately after the interview and at the time of this survey. Parents were asked if they felt they had benefited in any way by taking part and control parents were asked if they would have taken part without prior consent of their doctor. 90% of both cases and controls felt glad to have taken part immediately after the interview and few reported any anxiety at having done so; 95% of both cases and controls felt satisfied that they had made a worthwhile contribution. Although 18% of cases felt tense and 14% felt unhappy after the interview, over 90% of them felt glad that they had taken part a few weeks later. Of particular interest is that 38% of cases and 24% of controls said they had positively benefited from taking part in the UKCCS and 96% of control mothers did not mind their family doctor giving permission for them to be contacted. © 2001 Cancer Research Campaign http://www.bjcancer.com

**Keywords**: epidemiology; attitudes; ethics; participation; childhood cancer

It has been an on-going concern of researchers that in-depth questioning (whether by self-administered questionnaire or face-to-face interview) of individuals faced with certain illnesses, may cause distress (Carter and Deyo, 1981; Boring et al, 1984; Runeson and Beskow, 1991). The problem may be particularly acute when parents of children with potentially fatal diseases are asked questions which relate to their lifestyle such as smoking and alcohol intake. Members of ethics committees also raise these concerns, when asked to approve new research projects (Hershey, 1982; Eardley et al, 1991). In addition, there is the issue of approaching 'healthy' controls, or their parents, and administering health questionnaires, which may precipitate fears in people unaffected by the disease under investigation.

A reply to these concerns is that the opportunity for a structured discussion with a professional researcher, may have a beneficial effect by releasing some of the stress that cases or case parents may be experiencing (Funch and Marshall, 1981; Savitz et al, 1986; Taylor et al, 1991). It is also thought that the notion of 'being able to help in any way' in the fight against life-threatening disease is of value to both cases and controls.

Despite the importance of these issues, thorough literature searches reveal that little systematic research has been done, especially with male participants, or with parents on behalf of their children. In this study we investigate the reactions of some of the parents participating in the United Kingdom Childhood Cancer Study (UKCCS) to having taken part in the study. We also test the hypothesis that taking part in a study of childhood cancer does not increase the self-reported 'anxiety' of parents of healthy controls.

*Received 10 July 2000*
*Revised 16 October 2000*
*Accepted 16 October 2000*

*Correspondence to:* K Muir

### MATERIALS AND METHODS

### Main study: the UKCCS

The subjects approached in this study were parents who had already given interviews in the UKCCS. The methods are described elsewhere (UK Childhood Cancer Study Investigators, 2000). Briefly, the UKCCS took place in the whole of England, Wales and Scotland between 1991 and 1998, and was administered by regional teams. The aim was to interview the parents of every child developing cancer during a 5-year period, as well as parents of healthy control children matched by age and sex with the case children. Two controls were interviewed for each case.

The hypotheses are described elsewhere (UK Childhood Cancer Study Investigators, 2000) and were tested by conducting a face-to-face interview with both parents wherever possible (a few interviews were conducted by telephone), as well as collecting information from medical records after obtaining parental consent. Measurements of terrestrial gamma and radon exposure in the home were taken and EMF fields in homes and schools were measured. Blood samples were taken from case children and parents but not from controls.

The majority of case interviews were conducted in the child's home (a few took place in hospital) at a phase in their treatment approved by the case child's consultant. The control interviews were conducted at home as soon after the case interview as possible. The interviews with the mothers lasted on average $1\frac{1}{4}$ hours, and those with the fathers, 20 minutes. The parents were asked detailed questions regarding their past and present addresses and occupations, their health and that of all their children, together with details of smoking, alcohol consumption and family medical history.

### This study: 'Reactions to Interview'

A target of 300 case families and 300 control families was agreed, but this was later reduced to 200 in each group due to the withdrawal of one region from this study. The duration of the study was to be one year. Case parents and parents of first-choice controls were sent a questionnaire between 3 and 4 weeks after the UKCCS interview. The 4 regions that agreed to take part in this study were responsible for sending out questionnaires which were returned in pre-paid envelopes to the administrative centre in Nottingham. Reminders to non-responders were sent out 3 weeks after the initial posting date. Reminders were not sent to bereaved parents.

The questionnaire incorporated parts from that used by Taylor et al (1991) and took about 20 minutes to complete. The questions varied slightly between cases and controls, and between mothers and fathers, in order to reflect the original face-to-face questions in the childhood cancer study.

Information was collected about the reasons for agreeing to take part in the UKCCS, preconceived reasons for the child's illness (cases only) and any changes in these subsequent to the interview, general reactions to the interview and the procedures used, and whether any questions caused distress or difficulty. We asked about the parents' feelings immediately after the interviews and also 2 weeks later. We asked the control parents if they would have taken part in the study had approval not been given by their general practitioner, and whether they would be prepared in the future to take part in similar studies investigating other diseases such as heart disease, arthritis, meningitis in children, and systemic lupus erythematosus. All parents were asked if they felt they had benefited in any way by taking part in the childhood cancer study.

Reassurance was given about the confidentiality of this study, and that their original interviewer would not see the self-completed questionnaire.

Responses to questions were entered into a Microsoft Access database and then analysed using the Statistical Package for Social Sciences. Frequency tables were produced and in order to compare the responses of cases and controls, cross-tabulations were carried out with chi-square tests to look for statistically significant differences. Missing values were excluded from the analyses.

### RESULTS AND DISCUSSION

This study is unique in that this is the first time participants' views have been sought in a study focusing on the emotive subject of the health of their own children. It is worth noting that the UKCCS is one of the largest studies to date to investigate childhood cancer aetiology and was very detailed. It included, in addition to a very comprehensive interviewer administered questionnaire, the use of exposure measurements and the giving of a blood sample.

371 case parents (64%) and 380 control parents (66%) returned completed questionnaires. In all, 54% and 58% of case and control respondents respectively were female ($P$ = 0.272).

The majority of case parents and almost half of the control parents were 'very' interested in taking part in the UKCCS (Table 1). Only 10% of control parents recorded being 'not very' or 'not at all' interested in taking part. Such high interest amongst control parents reflects the emotivity of serious childhood disease and over 90% of control parents in this study indicated that this was indeed their reason for agreeing to take part. 76 (20%) said they specifically wanted to help in the UKCCS, 47 (13%) did so because of a recent personal health experience, 19 (5%) did not like to refuse and 10 (3%) were persuaded by other people. These answers were not mutually exclusive. 8 (2%) participants offered other reasons: 'possible help for children' (5), 'curious about reasons for study' (1), 'persistence of requests' (1) and 'aware of need for interview' (1). Well over 90% of controls would have agreed to participate if it had been a study of any of 4 other illnesses, including one that is not widely known (systemic lupus erythematosus). The largest response however was to 'meningitis in children' (99%). Only 10 cases, (3%) and 17 controls (5%) had been interviewed on a health study prior to the UKCCS.

Cases were less concerned than controls about the time taken up by the interview, about letting a stranger into their house and about giving out personal information (Table 1). This latter factor was of concern to a fifth of cases and a third of controls. More controls than cases thought that the information given about the study before the interview was sufficient and helpful.

Consultants were asked to explain the study to the parents of the children who were ill. 225 (63%) parents recalled this and 108 (14%) recalled their general practitioner explaining it. Of the case parents who did not recall their consultant explaining the study to them, almost three-quarters thought that the information given to them about the study before the interview was sufficient. Permission for inclusion in the study was sought from each control parent's general practitioner, but only 31% of control mothers reported having been contacted by their family doctor about the UKCCS.

**Table 1** Reactions of parents before the interview

| Interview topic | Cases ($n$ = 371) $n$ (%) | Controls ($n$ = 380) $n$ (%) | $\chi^2$ (df)[a], $P$ value |
|---|---|---|---|
| Interest in the study | | | |
| Very | 266 (71.7) | 181 (47.6) | |
| Moderately | 83 (22.4) | 161 (42.4) | $45.26_{(2)}$, <0.001 |
| Not at all/Not very | 22 (5.9) | 38 (10.0) | |
| Concerns about taking part | | | |
| The time taken | 19 (5.4) | 57 (17.5) | $25.83_{(1)}$, <0.001 |
| Letting stranger into home | 24 (6.9) | 71 (22.0) | $32.40_{(1)}$, <0.001 |
| Giving personal information | 70 (19.5) | 105 (30.6) | $11.63_{(1)}$, <0.001 |
| Information given beforehand | | | |
| Sufficient | 262 (80.4) | 291 (86.9) | $5.12_{(1)}$, <0.023 |
| Helpful | 253 (85.2) | 273 (93.2) | $9.96_{(1)}$, <0.002 |

[a] df, Degrees of freedom.

© 2001 Cancer Research Campaign

Of importance to the credibility of the data of the UKCCS, is the fact that apart from the questions on pregnancies and (for case parents) the health of their child who was ill, the majority of cases and controls did not have difficulty in answering major sections of the UKCCS questionnaire (Table 2). Despite having pre-circulated questions on past addresses and employment history, these questions did cause some difficulty experienced equally by case and control parents. 59 (16%) parents of cases found the questions on the health of their child difficult to answer and 144 (39%) found these questions upsetting.

20 case parents (6%) and 14 control parents (4%) reported that other questions were difficult or upsetting to answer; there was no difference between men and women. Fathers did not describe these topics but out of 18 case mothers who did, 8 said that questions on family medical history were 'difficult' or 'upsetting' to answer, as did two control mothers.

Responses to questions on feelings and attitudes after the interview show that 95% of both case and control parents felt 'satisfied' with their contribution to the UKCCS interview (Table 3). Only 6 case parents regretted taking part immediately afterwards but 3 of these later felt glad that they had done so (the other 3 did not respond to this question). There were significant differences in responses between case and control parents when asked about other feelings. Understandably, parents of case children were more likely to have experienced feelings of tension, unhappiness and anger after the interview, the most common reason being that they were reminded of their child's illness. One fifth of both case and control parents who felt this way said it was because they had difficulty in remembering things. A similar proportion of case and control parents experienced frustration at their inability to answer some questions.

Participants were also asked how they would describe themselves 5 years ago and now (data not shown). Of the 225 (60%) control parents who said that 5 years ago they would describe themselves as people who were not easily upset, 6% said that they would now describe themselves as people who were easily upset. This is in marked contrast to the 225 (61%) case parents who described themselves as not easily upset 5 years ago; one quarter of them said that they would now describe themselves as being easily upset.

Significantly more of the case parents felt 'very glad' at having taken part in the study. This was true immediately after the interview and even more so at the time of completing this postal survey. At least 90% of each group felt 'glad' or 'very glad' that they had taken part at both time points. Our results are similar to those found in epidemiological studies of adult cancers. For example, Savitz et al (1986) in a study of breast cancer found that 90% of participants were glad they had taken part. In their study of cervical cancer, Taylor et al (1991) observed over 95% of participants being 'somewhat' or 'very' glad to have taken part. Montazeri et al (1996) reported that 96% of patients (cases with lung cancer and controls with chronic respiratory disease) found that being interviewed was 'very' or 'quite' acceptable.

Few parents reported any anxiety at having taken part but this was more common among cases (6%). Very few control parents reported negative feelings, which allays concerns that administering a detailed health questionnaire to 'healthy' control participants may promote fears. Also reassuring, is the fact that although significantly more case parents (38%) said they had actually benefited from participating in the UKCCS, 24% of control parents said that they too had benefited in some way. The most common description (by both groups) of the benefit was that they felt that there was now a possibility of finding a cause, followed by a feeling of satisfaction that they had 'helped' with research. Kavanaugh (1997) in a study of perinatal loss found

**Table 2** During the interview: whether questions were difficult or upsetting

| Interview topic | Cases ($n$ = 371) $n$ (%) | Controls ($n$ = 380) $n$ (%) | $\chi^2$ (df)[a] | $P$ value |
|---|---|---|---|---|
| Past addresses | | | | |
| Difficult | 46 (12.6) | 53 (14.3) | $0.43_{(1)}$, | 0.51 |
| Upsetting | 11 (3.3) | 5 ( 1.5) | $2.14_{(1)}$, | 0.14 |
| Employment history | | | | |
| Difficult | 41 (11.3) | 41 (11.1) | $0.01_{(1)}$, | 0.91 |
| Upsetting | 3 ( 0.9) | 2 ( 0.6) | (Fishers Exact) | 1.00 |
| Own health | | | | |
| Difficult | 20 (5.6) | 12 (3.3) | $2.32_{(1)}$, | 0.13 |
| Upsetting | 6 (1.8) | 6 (1.9) | (Fishers Exact) | 0.80 |
| Pregnancies[b] | | | | |
| Difficult | 38 (19.5) | 24 (11.1) | $5.99_{(1)}$, | 0.02 |
| Upsetting | 32 (17.9) | 25 (12.7) | $1.60_{(1)}$, | 0.21 |
| Questions on smoking | | | | |
| Difficult | 8 (2.3) | 5 (1.4) | $0.79_{(1)}$, | 0.37 |
| Upsetting | 7 (2.2) | 1 (0.3) | (Fishers Exact) | 0.07 |
| Health of ill child[c] | | | | |
| Difficult | 59 (15.9) | NA | | |
| Upsetting | 144 (38.8) | NA | | |

[a]df, Degrees of freedom. [b]Women only. [c]Case parents only.

**Table 3** Reactions of parents after the interview

| | Cases ($n$ = 371) $n$ (%) | Controls ($n$ = 380) $n$ (%) | $\chi^2$ (df)[a] | $P$ value |
|---|---|---|---|---|
| Feelings at end of interview | | | | |
| Tense | 53 (18.1) | 20 (7.8) | $12.0_{(1)}$, | < 0.001 |
| Unhappy | 39 (13.5) | 5 (2.0) | $23.0_{(1)}$, | < 0.001 |
| Satisfied with contribution | 340 (95.0) | 344 (95.6) | $0.0_{(1)}$, | 0.85 |
| Angry | 14 (5.0) | 3 (1.2) | $5.1_{(1)}$, | 0.025 |
| Frustrated at inability to answer | 115 (38.3) | 85 (30.7) | $3.5_{(1)}$, | 0.061 |
| Attitudes to having taken part in the study: Immediately afterwards | | | | |
| Very glad | 162 (44.6) | 111 (30.1) | | |
| Glad | 167 (46.0) | 221 (59.9) | | |
| No strong feelings | 28 (7.7) | 35 (9.5) | $19.94_{(3)}$, | < 0.001 |
| Regretted it | 6 (1.7) | 2 (0.5) | | |
| At the time of the survey | | | | |
| Very glad | 187 (53.7) | 124 (35.7) | | |
| Glad | 143 (41.1) | 192 (55.3) | | |
| No strong feelings | 18 (5.2) | 31 (8.9) | $24.92_{(3)}$, | < 0.001 |
| Regretted it | 0 (0.0) | 1 (0.3) | | |
| The interview... | | | | |
| Caused anxiety? | 22 (6.0) | 8 (2.1) | $7.39_{(1)}$, | 0.007 |
| Benefited you? | 136 (38.4) | 87 (23.6) | $18.56_{(1)}$, | < 0.001 |
| Would you recommend a friend to take part? | | | | |
| Yes | 314 (86.5) | 284 (77.2) | | |
| Not advise | 36 (10.0) | 78 (21.2) | $19.96_{(2)}$, | < 0.001 |
| No | 13 (3.6) | 6 (1.6) | | |

[a]df, Degrees of freedom.

© 2001 Cancer Research Campaign that most parents felt participation helpful or at least innocuous. In their paper categorizing participant benefit, Hutchinson et al (1994) describe this type of benefit as giving participants a ‘sense of purpose’ and describe the good feeling they derive when sharing information with researchers that may in turn be shared with other professionals or lay people through publication. A quarter of control parents who gave a description of the benefit said that they were now more aware of how healthy they were. The majority of participants would recommend a friend to take part in this study.

Various procedural strategies were incorporated in the UKCCS to minimize distress to case parents. These included giving sufficient information before the interview, and asking the consultant to explain the study to the parents and to indicate appropriate timing for the interview in relation to the course of the child’s illness. Timing of interview was considered to be ‘about right’ by 89% of parents, 2% thought it ‘too soon’ and 9% thought it ‘too late’, the proportions being similar in mothers and fathers. Most interviews took place in the parents’ home (92% and 98% of case and control parents, respectively) and all but 6 case parents and 1 control parent were happy about this. Very few parents (8%) would have preferred a postal questionnaire and most of these were controls. An important ethical question has also been answered in this study in that 96% of the control mothers and 97% of control fathers did not mind their doctor giving permission for them to be contacted by the UKCCS interviewer. Over half of the fathers and 44% of the mothers said that they would have given an interview without their doctor’s consent being sought. A similar result was found by Taylor et al (1991).

The results of this study show support for well organized studies which focus on vulnerable populations and sensitive topics and offer reassurance to future study organizers, participants and ethics committees alike.

### ACKNOWLEDGEMENTS

We are grateful to the United Kingdom Childhood Cancer Study for access to study participants. The UKCCS is conducted by 12 teams of investigators (10 clinical and epidemiological and 2 biological) based in university departments, research institutes and the National Health Service in Scotland. The work is co-ordinated by a Management Committee and in Scotland by a Steering Group of paediatric oncologists and by the National Radiological Protection Board. Funding is provided by a consortium of statutory bodies, cancer charities and industrial sponsors.

We thank the staff from the following Regions that participated in the follow up study: South West, North West, Trent and East Anglia, and in particular: Sue Muir, Judi Paxon and Emmanuel Ruja. We thank Penny Kelham for her invaluable help in preparing the questionnaire and Simon Hawtin for data entry. We thank Dr Peter Maguire of the CRC Psychological Medicine Group for his advice. We especially thank the parents who agreed to help with this additional study.

### REFERENCES

Boring CC, Brockman E, Causey N, Gregory HR and Greenberg RS (1984) Patient attitudes towards physician consent in epidemiologic research. *Am J Pub Hlth* **74**: 1406–1408

Carter WB and Deyo RA (1981) The impact of questionnaire research on clinical populations: A dilemma in review of human subjects research resolved by a study of a study. *Clin Res* **29**: 287–295

Eardley A, Cribb A and Pendleton L (1991) Ethical issues in psychological research among patients with cancer. *Eur J Cancer* **27**: 166–169

Funch DP and Marshall JR (1981) Patient attitudes following participation in a health outcome survey. *Am J Pub Hlth* **71**: 1396–1398

Hershey N (1982) Putting the lamentations of epidemiologists in perspective. *Am J Pub Hlth* **72**: 1155–1157

Hutchinson SA, Wilson ME and Wilson HS (1994) Benefits of participating in research interviews. *J Nursing Scholarship* **2**: 161–164

Kavanaugh K (1997) The parental experience surrounding the death of an infant born at the margin of viability. *J Obstet, Gynaecol Neonatal Nursing* **26**: 43–51

Montazeri A, Milroy R, Gillis CR and McEwen J (1996) Interviewing cancer patients in a research setting: the role of effective communication. *Support Care Cancer* **4**: 447–454

Runeson J and Beskow J (1991) Reactions of survivors of suicide victims to interviews. *Acta Psychiat Scand* **83**: 169–173

Savitz DA, Hamman RF, Grace C and Stroo K (1986) Respondents’ attitudes regarding participation in an epidemiologic study. *Am J Epidemiol* **123**: 362–366

Taylor C, Trowbridge P and Chilvers C (1991) Stress and cancer surveys: attitudes of participants in a case-control study. *J Epidemiol Comm Hlth* **45**: 317–320

UK Childhood Cancer Study Investigators (2000) The United Kingdom Childhood Cancer Study: objectives, materials and methods. *Br J Cancer* **82**: 1073–1102

© 2001 Cancer Research Campaign
